# M1 Macrophage-Derived Exosomes Loaded with Gemcitabine and Deferasirox against Chemoresistant Pancreatic Cancer

**DOI:** 10.3390/pharmaceutics13091493

**Published:** 2021-09-17

**Authors:** Yongmei Zhao, Yuanlin Zheng, Yan Zhu, Yi Zhang, Hongyan Zhu, Tianqing Liu

**Affiliations:** 1 School of Pharmacy, Nantong University, Nantong 226019, China; ymzhao@ntu.edu.cn (Y.Z.); 2019310034@stmail.ntu.edu.cn (Y.Z.); 2019320002@stmail.ntu.edu.cn (Y.Z.); amy600@ntu.edu.cn (H.Z.); 2 School of Chemistry, University of Glasgow, Glasgow G12 8QQ, UK; 2449900z@student.gla.ac.uk; 3 NICM Health Research Institute, Western Sydney University, Sydney, NSW 2145, Australia

**Keywords:** exosomes, gemcitabine, deferasirox, pancreatic cancer, chemoresistance, RRM2

## Abstract

Pancreatic cancer is a malignant disease with high mortality and poor prognosis due to lack of early diagnosis and low treatment efficiency after diagnosis. Although Gemcitabine (GEM) is used as the first-line chemotherapeutic drug, chemoresistance is still the major problem that limits its therapeutic efficacy. Here in this study, we developed a specific M1 macrophage-derived exosome (M1Exo)-based drug delivery system against GEM resistance in pancreatic cancer. In addition to GEM, Deferasirox (DFX) was also loaded into drug carrier, M1Exo, in order to inhibit ribonucleotide reductase regulatory subunit M2 (RRM2) expression via depleting iron, and thus increase chemosensitivity of GEM. The M1Exo nanoformulations combining both GEM and DFX significantly enhanced the therapeutic efficacy on the GEM-resistant PANC-1/GEM cells and 3D tumor spheroids by inhibiting cancer cell proliferation, cell attachment and migration, and chemoresistance to GEM. These data demonstrated that M1Exo loaded with GEM and DFX offered an efficient therapeutic strategy for drug-resistant pancreatic cancer.

## 1. Introduction

Pancreatic cancer is a lethal disease with poor survival rate and an increasing incidence due to lack of early diagnosis and low treatment efficiency after diagnosis [1]. As less than 20% of patients are suitable for resection, chemotherapy is still one of the main treatments for pancreatic cancer [2,3]. However, current treatment strategies are still unsatisfactory and have failed to significantly increase the overall survival time of patients with pancreatic cancer over the last decade.

Gemcitabine (GEM), a hydrophilic deoxycytidine analogue, is a first-line chemotherapeutic drug and is widely used in the treatment of unresectable pancreatic cancer. However, chemoresistance is still the major problem that limits the therapeutic efficacy of GEM. The positive response rate for standard GEM treatment in pancreatic cancer patients is only 6% [4]. Although not completely understood, the mechanism of GEM resistance is associated with regulation of drug transport, DNA damage and repair, and renewability of cancer stem cells [5,6,7,8,9,10]. Ribonucleotide reductase (RR) is an enzyme that catalyzes the formation of deoxyribonucleotides from ribonucleotides, which is essential for cell replication. This enzyme contains two subunits, M1 (RRM1) and M2 (RRM2). It has been reported that RRM2 plays an essential role in cancer cell proliferation and the development of resistance to GEM in pancreatic tumor cells [11,12]. Studies have demonstrated that overexpression of RRM2 enhanced DNA damage repair and replication, leading to decreased chemosensitivity of GEM [13]. In addition, clinical data demonstrated that patients who have elevated RRM2 expression had less response to gemcitabine-based chemotherapy, and high expression of RRM2 in pancreatic cancer is associated with a poor prognosis [14]. Therefore, RRM2 has become a potential target to overcome GEM resistance. Iron chelators have been identified as RRM2 inhibitors as they can interact with the essential diiron tyrosyl radical center and inhibit the enzyme activity. Deferasirox (DFX) is an oral iron chelator for the treatment of iron overload. Studies have demonstrated that DFX can significantly downregulate the expression of RRM2 and potentiate the therapeutic effects of GEM, resulting in improved anticancer efficacy [15]. However, the application against GEM-resistant cancer is limited due to variable bioavailability and low intratumoral distribution [16,17]. Therefore, there is an urgent need to develop advanced delivery strategies to overcome these issues.

Exosomes are nanosized (30–150 nm) extracellular vesicles secreted by cells for intercellular communications. They are promising natural drug carriers with advantages including excellent biocompatibility, long circulation, and low immunogenicity [18,19,20]. Moreover, exosomes as delivery vesicles can fuse with target cells and directly transport loaded drugs to receptor cells in order to overcome p-glycoprotein-involved drug resistance [21]. In addition, exosomes derived from different cell types contain cell-specific bioactive lipids, proteins, and genetic materials, which allows them to have the specific biological functions [22]. Macrophages are a key immune cell population involved in the tumor microenvironment and can be polarized into M1 or M2 phenotypes in response to different stimuli. M1 macrophages produce high levels of proinflammatory and immunostimulatory cytokines, including interleukin 12 (IL-12), interleukin 23 (IL-23), tumor necrosis factor alpha (TNFα) etc., leading to tumor suppression [23]. It has been reported that M1 macrophage-derived exosomes (M1Exo) can release pro-inflammatory signals and generate a stimulatory tumor immune-microenvironment, implying that they have great therapeutic potentials for anti-cancer therapy [24]. More recently, M1Exo has been used as drug carrier to deliver paclitaxel into the tumor tissue, and results demonstrated that M1Exo provided a pro-inflammatory environment which further enhanced the therapeutic efficacy of chemotherapy by activating the apoptosis pathway [25]. Similar findings were presented by Li and Wang et al., showing that anti-tumor efficiency of M1Exo loaded with cisplatin was significantly improved in vivo via upregulating Bcl-2-associated X protein (Bax) and caspase-3 in the apoptosis pathway [26]. Therefore, engineered M1Exo can be a promising approach for drug delivery in cancer therapy.

In this study, M1Exo was engineered as drug carrier to co-delivery DFX and GEM to overcome the chemoresistance of GEM and improve its therapeutic potential. Our aim is to achieve efficient delivery of DFX and at the same time sensitize GEM-resistant pancreatic cancer cells to this chemotherapy. Our results show that the M1Exo-based co-delivery of DFX and GEM can be used as a promising strategy for drug-resistant pancreatic cancer treatment via the inhibition of cancer cell proliferation, metastasis, and chemoresistance.

## 2. Materials and Methods

### 2.1. Cell Culture

Human monocyte THP-1 cells and human pancreatic cancer PANC-1 cells were purchased from National Collection of Authenticated Cell Cultures, China. THP-1 cells were maintained in Roswell Park Memorial Institute medium (RPMI 1640, Thermo Fisher Scientific, Waltham, MA, USA) supplemented with 10% fetal bovine serum (FBS, Thermo Fisher Scientific) and 1% penicillin-streptomycin (PS, Thermo Fisher Scientific). PANC-1 cells were cultured in Dulbecco’s Modified Eagle Medium (DMEM, Thermo Fisher Scientific) supplemented with 10% FBS and 1% PS. THP-1 were differentiated to macrophage by culturing in serum-free RPMI1640 medium with 100 ng/mL phorbol ester (PMA, Sigma-Aldrich, New York, NY, USA) and 0.3% bovine Serum Albumin (BSA, Sigma-Aldrich) for 72 h. GEM-resistant pancreatic cancer cells (PANC-1/GEM) were induced by treatment with 18 µg/mL GEM (Sigma-Aldrich) for ten months. Both PANC-1 and PANC-1/GEM were cultured in a humidified incubator containing 5% CO_2_ at 37 °C. 

### 2.2. Exosome Isolation and Characterization 

The TPH-1 differentiated macrophages (M0 phenotype) were seeded in 6-well plate at the density of 10^6^ cells/mL. After 24 h incubation, cells were stimulated with 100 ng/mL lipopolysaccharide (LPS, Sigma-Aldrich) for 24 h to induce M1 macrophage polarization [27]. Next, the culture media of M1 macrophage were collected in order to obtain M1Exo. The media were centrifugated at 800× *g* (10 min), 3000× *g* (10 min), and 10,000× *g* (30 min) at 4 °C to remove cell debris and large extracellular vesicles. M1Exo were then harvested by ultracentrifugation at 100,000× *g* for 70 min at 4 °C using an ultracentrifuge (Optima XPN-100, Beckman Coulter, Indianapolis, IN, USA) [28]. 

The morphology of the exosomes was characterized using a transmission electron microscope (TEM, Talos F200X, Thermo Fisher Scientific, Waltham, MA, USA). In brief, a drop of the isolated exosomes in PBS solution was added onto carbon-coated copper grids (Sigma-Aldrich). After drying for 5 min at room temperature, the samples were stained with 1% uranium acetate (Sigma-Aldrich) for 1 min and the excess solution was removed via a filter paper. The samples were further dried for 20 min at room temperature and then imaged by TEM, and the TEM particle size data were converted directly to cumulative number-based distributions. Particle size and zeta potential of the exosomes were measured by dynamic light scattering (DLS, Nano-zs30, Malvern Panalytical, Malvern, UK). 

### 2.3. Drug Loading and Quantification

The exosomes (100 µg) and drug mixture (100 µg) were mixed in 400 µL of PBS solution, and electroporated in 4mm path length electroporation cuvettes using a Bio-Rad electroporation instrument. The electroporation was performed at a voltage of 400 V and an electric capacity of 150 mF with 1 ms of discharging time. The mixture was then incubated at 37 °C for 30 min to recover the exosome membrane [29]. The un-encapsulated GEM and DFX (Sigma-Aldrich) was removed by passing through an amicon filter (100 kDa, Merk Millipore, Burlington, VT, USA) and centrifuged at 100,000× *g* for 60 min. 

The drug loading efficiency in the exosomes was measured by dissolving the exosomes with methanol to completely release GEM and DFX, and the released GEM and DFX were quantified by a high-performance liquid chromatography with ultraviolet detection (HPLC-UV) (Agilent Scientific Instruments, Santa Clara, CA, USA) at wavelengths of 275 nm and 245 nm, respectively.

### 2.4. In Vitro Cell Viability

The cellular anti-cancer effects of M1Exo loaded with GEM and DFX (M1Exo-GEM-DFX) against both PANC-1 and PANC-1/GEM cells were evaluated by MTS (3-(4,5-dimethylthiazol-2-yl)-5-(3 carboxymethoxyphenyl)-2-(4-sulfophenyl)-2H-tetraz olium) assay (Sigma-Aldrich). Cells (1 × 10^4^ cells/well) were seeded into 96-well plates. After 12 h incubation, the cells were treated with control, M1Exo, GEM, DFX, GEM&DFX, and M1Exo-GEM-DFX at corresponding concentrations of GEM (18 µg/mL) and DFX (18 µg/mL) for 48 h at 37 °C and 5% CO_2_. After incubation, the culture medium in each well was replaced with 100 µL of MTS solution (20 µL CellTiter 96^®^ AQueous One Solution Reagent and 80 µL tissue culture medium). After 1 h incubation, the absorbance was detected at 490 nm using a microplate reader (Thermo Fisher Scientific, Waltham, MA, USA) to investigate the cell viability.

### 2.5. Iron Removal Efficacy Study

Both PANC-1 and PANC-1/GEM cells were treated with PBS control, M1Exo, GEM, DFX, GEM&DFX, and M1Exo-GEM-DFX for 24 h. The cells were then harvested using Trypsin (Thermo Fisher Scientific), washed twice with PBS, and counted in a cell counting chamber. The final cell pellets were collected and digested, and their iron content was determined using inductively coupled plasma mass spectrometry (ICP-MS, Agilent 7700, Agilent Scientific Instruments, Santa Clara, CA, USA) under routine element operating conditions.

### 2.6. Multidrug Resistance (MDR) Study

Multidrug resistance study was performed by assessing drug efflux activity using the Vybrant Multidrug Resistance Assay (Thermo Fisher Scientific) following the manufacturer’s instructions. PANC-1/GEM cells could express high levels of drug transporter, p-glycoprotein, that rapidly eliminates nonfluorescent calcein AM from the plasma membrane and reduces the intake of fluorescent calcein in the cytosol. Therefore, the p-glycoprotein activity can be quantitated by measuring the accumulation of intracellular calcein fluorescence. In brief, the cells were seeded on 12-well plates and treated with verapamil (Thermo Fisher Scientific), control calcein (Thermo Fisher Scientific), and other drug treatments including M1Exo, GEM, DFX, GEM&DFX, and M1Exo-GEM-DFX. The calcein retention was measured using a microplate reader, with calcein-specific fluorescence absorption maximum at 494 nm and the emission maximum at 517 nm.

### 2.7. Wound Healing Assays

Wound healing study was carried out using our method as previously described [30]. In brief, cells were seeded in 96-well plates and cultured until they reached more than 80% confluence and incubated in serum-free medium to eliminate the effect of cell proliferation. The wounds on the cell monolayers were generated using an Incucyte wound maker. The cells were then treated with fresh serum-free medium containing drug-loaded M1Exo or controls, their wound healing behaviors were monitored using Incucyte Zoom (Essen BioScience, Ann Arbor, MI, USA) and analyzed with IncuCyte Zoom software (IncuCyte® Scratch Wound Cell Migration Software Module, BioScience, Ann Arbor, MI, USA).

### 2.8. Cell Attachment Study

PANC-1/GEM cells were seeded onto 6-well plates and cultured until they reached more than 80% confluence. The cells were treated with drug-loaded M1Exo or controls for 24 h. After being washed with PBS and detached using versine, 5 × 10^4^ cells per well were seeded onto gelatine precoated 96-well plates for 2 h and then fixed with 4% paraformaldehyde (PFA, Thermo Fisher Scientific) for 15 min. The attached cells were imaged using Incucyte Zoom (Essen) and the number of cells was quantified using IncuCyte Zoom software.

### 2.9. Western Blotting

The expression of both RRM2 and equilibrative nucleoside transporter-1 (hENT1) markers were assessed by Western blotting. Cells were treated with drug-loaded nanoparticles or controls for 24 h, and washed with PBS. Total protein was harvested and quantified using the bicinchoninic acid protein assay kit (Thermo Fisher Scientific). Protein samples were prepared at a concentration of 50 μg protein/20 μL. Samples in loading buffer were heated at 75 °C for 10 min, loaded into wells of 15% acrylamide gels (Thermo Fisher Scientific) with a protein ladder, and run on SDS-PAGE at 100 V. The proteins were subsequently transferred onto nitrocellulose membranes (Thermo Fisher Scientific) for 1 h and blocked at 4 °C overnight. Blots were treated with an anti-RRM2 antibody (1:1000, Abcam) or an anti-hENT1 antibody (1:1000, Abcam) in blocking buffer for 1 h at room temperature on a shaker, while an anti-β-actin monoclonal antibody was a loading control (1:1000, Abcam). After washing with Tris Buffered Saline with Tween (TBST, Thermo Fisher Scientific) 4 times, the blots were incubated with secondary antibodies (1:5000, Abcam) in the dark for 1 h. After washing 4 times and drying, the membranes were scanned with the Odyssey imaging system (LI-COR) (LI-COR Biosciences, Lincoln, Dearborn, MI, USA).

### 2.10. Inhibition of 3D PANC-1 and PANC-1/GEM Tumor Spheroids 

PANC-1/GEM tumor spheroids were formed in microwell devices using our reported method [31,32,33]. The culture medium was changed every 2 days and the formation of the tumor spheroids was monitored using a light microscope (OLYMPUS, CKX53,Tokyo, Japan). When the size of the tumor spheroids reached 200 μm in diameter, the spheroids were treated with drug-loaded nanoparticles or controls at a concentration of 50 µg/mL of GEM or of 50 µg/mL of DFX, respectively, for 7 days. The growth of the spheroids was recorded using a light microscope and the roundness was calculated using the following formula: roundness (%) = 100 − (R − r)/R × 100 (R: represents the radius of the minimum circumscribed circle; r: represents the maximum inscribed concentric circle) and analyzed by imageJ [34]. In addition, the tumor spheroid volume was calculated with the following formula: V = (π × d_max_ × d_min_)/6 and the change ratio of the tumor spheroid volume was compared with initial volume of each group [35]. 

### 2.11. Statistical Analysis

Multiple group comparisons were carried out using t-tests using GraphPad Prism version 9 (GraphPad, San Diego, CA, USA). All results are shown as mean ± the standard deviations of at least three replicates. *p* < 0.05 was considered to be significantly different.

## 3. Results and Discussion

### 3.1. M1Exo Preparation, Characterization, and Drug Loading

**Figure 1:**
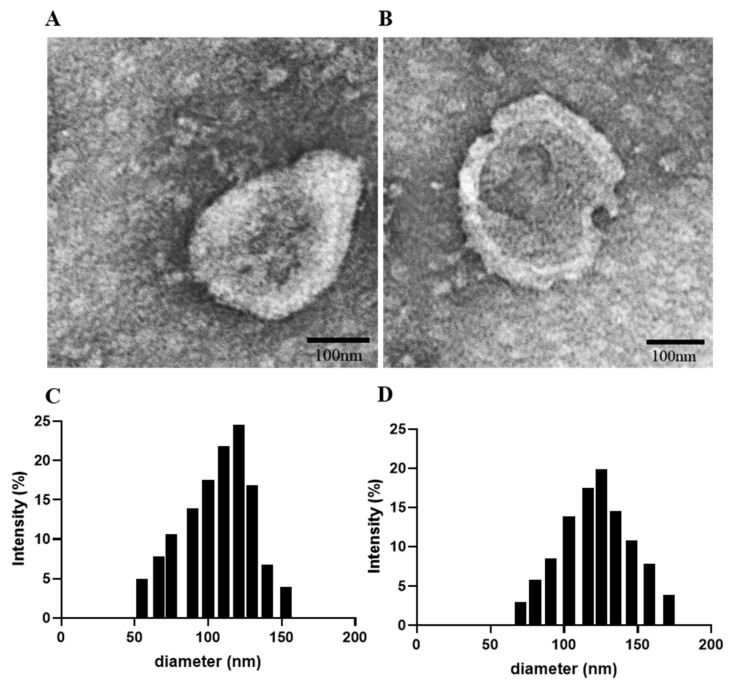
Characterization of M1Exo with or without drug loading: TEM images of (**A**) M1Exo and (**B**) M1Exo-GEM-DFX and the TEM particle size data which were converted directly to cumulative number-based distributions, (**C**) M1Exo, and (**D**) M1Exo-GEM-DFX.

TPH-1-derived macrophages were polarized using cytokines IFN-γ or LPS for 24 h until they turned into M1 phenotypes. M1Exo were collected and loaded with both DFX and GEM via electroporation. M1Exo with or without drug loading were characterized by morphology, hydrodynamic size, surface charge, and loading efficiency. The morphology of M1Exo and M1Exo-GEM-DFX was measured by TEM and showed that they were a uniform spherical shape with narrow size distribution (Figure 1). The hydrodynamic size of M1Exo and M1Exo-GEM-DFX was investigated by DLS analysis. The results showed that the size of M1Exo was 120.1 ± 0.5 nm, while the size increased to 150.9 ± 1.1 nm after encapsulation of GEM and DFX (Table 1). The loading efficiency was measured by HPLC via generating a standard curve at 275 nm and 245 nm for GEM and DFX, respectively. The encapsulation efficiency was around 6.5 ± 2.3 and 5.7 ± 1.4 for GEM and DFX, respectively, as shown in Table 1.

### 3.2. In Vitro Cell Viability Study

**Figure 2:**
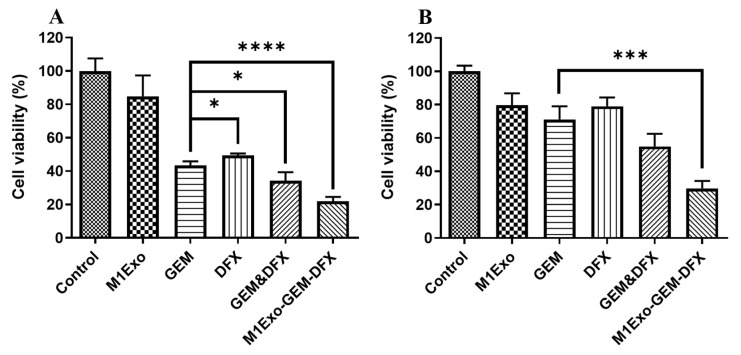
Cell viability study determined by the MTS assay with treatment of different drug formulations, including M1Exo, GEM, DFX, GEM&DFX, and M1Exo-GEM-DFX at a corresponding concentration of GEM and DFX in (**A**) PANC-1 cells and (**B**) PANC-1/GEM cells. (* *p* < 0.05, *** *p* < 0.001, **** *p* < 0.0001).

Both PANC-1 and drug-resistant PANC-1/GEM cells were used to investigate the cytotoxicity effects of M1Exo-GEM-DFX. Based on the viability study using MTS assays, native empty M1Exo did not show significant cytotoxic effects on both PANC-1 and PANC-1/GEM cells (Figure 2). Although both free GEM and free DFX showed greater cytotoxicity to PANC-1 cells compared with the control group (Figure 2A), neither of them had an obvious cytotoxicity effect on PANC-1/GEM cells (Figure 2B). The cell viability of the GEM&DFX group against PANC-1 cells was around 33%, while higher cell viability (~55%) was observed when treated with PANC-1/GEM cells. These results indicated that free drugs including GEM, DFX, and GEM&DFX was able to inhibit the proliferation of PANC-1 cells, however, they had less or no cytotoxic effect on drug-resistant PANC-1/GEM cells. In contrast, M1Exo-GEM-DFX showed greatest inhibition of cell viability against both PANC-1 cells and PANC-1/GEM cells. Moreover, the cell viability of M1Exo-GEM-DFX against PANC-1/GEM cells was around 29%, which was significantly lower than other groups (Figure 2B), suggesting that M1Exo-GEM-DFX has great potential to reverse drug resistance for pancreatic cancer treatment.

### 3.3. Iron Removal Efficacy

**Figure 3:**
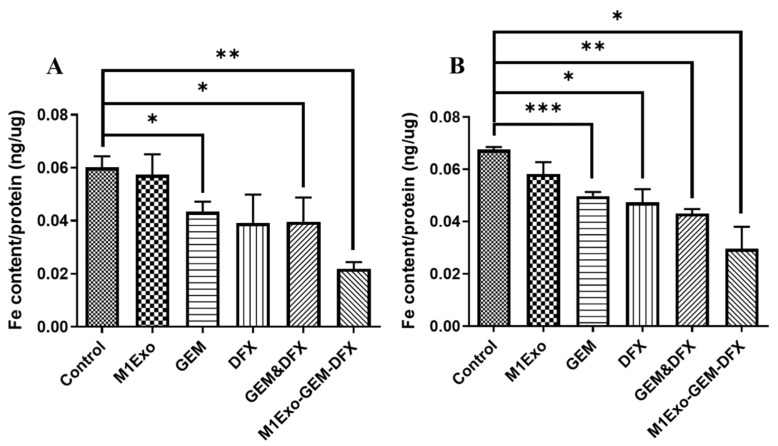
Iron removal efficacy of M1Exo, GEM, DFX, GEM&DFX, and M1Exo-GEM-DFX at a corresponding concentration of GEM and DFX in (**A**) PANC-1 cells and (**B**) PANC-1/GEM cells. (* *p* < 0.05, ** *p* < 0.01, *** *p* < 0.001).

To understand whether M1Exo-GEM-DFX could efficiently remove iron from both PANC-1 and drug-resistant PANC-1/GEM cells, we measured the iron content after the treatment with the drug formulations. It was observed that native empty M1Exo did not show iron-removing ability on PANC-1 or PANC-1/GEM cells (Figure 3). GEM, DFX, and GEM&DFX slightly reduced the iron amount in PANC-1 cells compared with the control group (Figure 3A), whereas, in Figure 3B, their iron-removing ability decreased during the treatment of PANC-1/GEM cells. In addition, we found that the iron content of either PANC-1 or PANC-1/GEM cells treated with M1Exo-GEM-DFX was the lowest compared with other groups, indicating that M1Exo-GEM-DFX had the best iron removal efficacy, even in drug-resistant cells. This novel formulation can be an effective iron chelation strategy for cancer treatment.

### 3.4. Expression of Drug Resistance-Related Proteins

**Figure 4:**
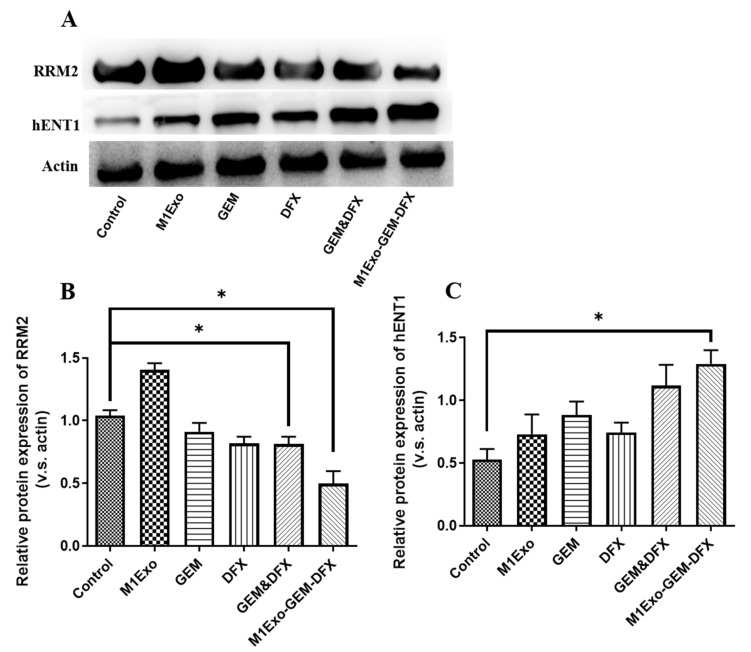
(**A**) Western blot of RRM2 and hENT1 expression in PANC-1/GEM cells at 24 h post-transplant. Figure 1. Exo, GEM, DFX, GEM&DFX, and M1Exo-GEM-DFX, while β-actin was used as internal control. (**B**) Relative expression level of RRM2. (**C**) Relative expression level of hENT1. (* *p* < 0.05).

To investigate the mechanism and the degree of GEM resistance after the M1Exo-GEM-DFX treatments, the protein expression of RRM2 and hENT1 was detected using Western blot analysis. In Figure 4A,B, the band intensity of RRM2 of the M1Exo-GEM-DFX group was the lowest among all the treatment groups, suggesting the expression of the RRM2 protein was significantly downregulated in the cells treated with M1Exo-GEM-DFX. This reduced RRM2 expression is mainly related to the low iron supply induced by the iron chelator as hENT1 is the main transporter of GEM to penetrate the cell membrane [36]. We then measured the expression of hENT1 and found that the expression of hENT1 was significantly increased in the M1Exo-GEM-DFX group compared with other groups (Figure 4A,C). Taken together, these data demonstrated that M1Exo-GEM-DFX not only inhibited the RRM2 expression via iron depletion, but also promoted the hENT1 expression to efficiently transport the drug combinations into cells. Thus, M1Exo-GEM-DFX was able to overcome GEM resistance, which may lead to better therapeutic outcomes for patient with GEM-resistant pancreatic cancer.

### 3.5. Anti-MDR Effects

**Figure 5:**
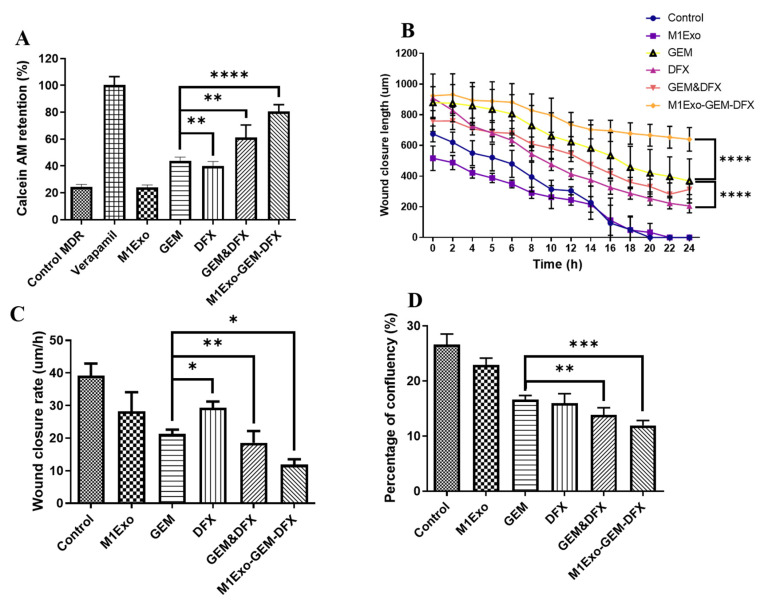
(**A**) Anti-MDR study of M1Exo, GEM, DFX, GEM&DFX, and M1Exo-GEM-DFX against PANC-1/GEM compared with blank and positive controls. Wound-healing study of M1Exo, GEM, DFX, GEM&DFX, and M1Exo-GEM-DFX against PANC-1/GEM: (**B**) Wound-healing assay of wound closure length. (**C**) Wound-closure rate. (**D**) Percentage of confluency measured by cell attachment assay. (* *p* < 0.05, ** *p* < 0.01, *** *p* < 0.001, **** *p* < 0.0001).

Overexpression of P-glycoprotein (p-gp) can lead to MDR in many cancers due to its ability to efflux intracellular anticancer drugs. Therefore, inhibiting p-gp is a promising strategy to improve the chemosensitivity of GEM in PANC-1/GEM cells. As shown in Figure 5A, both M1Exo and the blank control had relatively low level of calcein AM retention, while the calcein AM retention levels were increased for both GEM and DFX treatment groups. Meanwhile, when treated with M1Exo-GEM-DFX, the calcein AM retention level was over 80% compared to positive control verapamil, a classic P-gp inhibitor. These results indicated that M1Exo-GEM-DFX treatment efficiently suppressed the expression of P-gp in PANC-1/GEM cells, leading to increased accumulation of chemotherapeutic agents in the cytosol.

### 3.6. Cell Migration and Attachment

Cell migration and attachment plays a critical role in cancer cell invasion and tumor metastasis. Thus, we used wound-healing assay to investigate cell migration ability in vitro, which is a commonly used model to mimic cancer cell metastasis. As shown in Figure 5B,C, both wound length and rate of wound closure for PANC-1/GEM treated with M1Exo-GEM-DFX remain unchanged when compared to other treated groups. In Figure 5D, GEM, DFX, GEM&DFX treatment groups had slightly reduced cellular confluency compared to control and M1Exo groups. In contrast, PANC-1/GEM cells which received M1Exo-GEM-DFX treatment had the lowest cellular confluency during cell attachment assay due to its reduced adhesion ability of PANC-1/GEM cells. These results suggest that M1Exo-GEM-DFX dramatically inhibited the invasiveness of GEM-resistant pancreatic cancer cells.

### 3.7. Tumor Spheroid Assay

**Figure 6:**
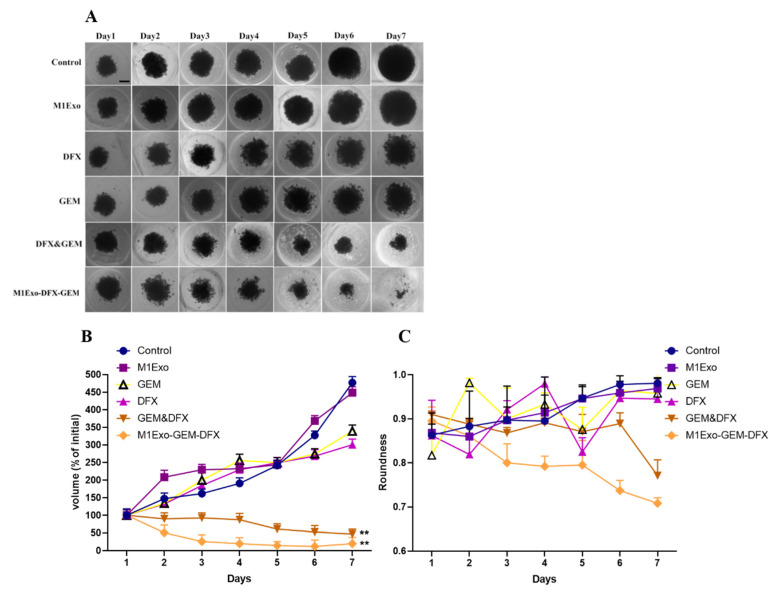
(**A**) Morphology of 3D PANC-1/GEM tumor spheroids treated with blank control, M1Exo, GEM, DFX, GEM&DFX, and M1Exo-GEM-DFX at the indicated concentration for 7 days. Scale bar: 200 µm. (**B**) Inhibitory effect on the growth of PANC-1/GEM tumor spheroids. (**C**) PANC-1/GEM tumor spheroid roundness after treatments, calculated by ImageJ. (** *p* < 0.01).

The anticancer effect was further evaluated in a 3D tumor spheroid system which has been widely used for anticancer drug screening due to better mimicking the physiological properties of tumor tissue [37]. Here, PANC-1/GEM tumor spheroids were successfully cultured to evaluate the potential anti-tumor activity. Figure 6A represents the inhibitory effects of the applied formulations on the 3D PANC-1/GEM tumor spheroids. As shown in Figure 6B, native empty M1Exo had no effect on inhibiting the volume of the PANC-1/GEM tumor spheroids, as a solid cellular cluster structure was maintained after the treatment. Meanwhile, the tumor spheroid formation efficiency of both GEM&DFX and M1Exo-GEM-DFX decreased compared with other treatment groups in PANC-1/GEM tumor spheroids. In addition, the volume change ratio of tumor spheroids at day seven was 477.2 ± 17.2%, 448.5 ± 16.8%, 339.3 ± 17.3%, 300.6 ± 15.9%, 47.1 ± 14.1% and 20 ± 17.6% for control, M1Exo, GEM, DFX, GEM&DFX, and M1Exo-GEM-DFX, respectively. Among these formulations, M1Exo-GEM-DFX induced the strongest inhibitory effect on PANC-1/GEM tumor spheroid growth. The measurement of the spheroid roundness was compared, and we found that the tumor spheroids became granular and irregular on the periphery and finally broke into pieces when treated with M1Exo-GEM-DFX (Figure 6C). These results were consistent with the previous results of cell migration and attachment study. Our results indicated that M1Exo-GEM-DFX effectively inhibited the formation and growth of PANC-1/GEM tumor spheroids compared with free drugs. Thus, these data provided solid evidence that M1Exo-GEM-DFX can significantly enhance therapeutic efficacy toward drug-resistant PANC-1/GEM in vitro by inducing PANC-1/GEM cell death and increasing drug sensitivity to GEM.

## 4. Conclusions

In summary, co-delivery of gemcitabine and Deferasirox using M1Exo offers an effective solution for treating drug-resistant pancreatic cancer. Our study revealed that M1Exo-GEM-DFX nanoformulation enhanced the cytotoxicity efficacy on the GEM-resistant PANC-1/GEM cell line. The mechanism of action was associated with increasing chemosensitivity of GEM in PANC-1/GEM cells by inhibition of RRM2 expression via depleting iron. We also investigated the anticancer therapeutic efficacy of the nanoformulations using a 3D tumor spheroid model. We demonstrated that M1Exo-GEM-DFX effectively inhibited the formation and growth of PANC-1/GEM tumor spheroids compared with free drugs. Overall, the present study suggested that M1Exo-GEM-DFX could be an efficient therapeutic strategy for the treatment of drug-resistant pancreatic tumors and provided insight into their mechanism of action.

## Figures and Tables

**Table 1:** Physical properties and drug loading efficiency of M1Exo formulations. ^a^ Determined by DLS.

Sample	Hydrodynamic Size D_h_^a^ (nm)	Zeta-Potential (mV)	Drug Loading (%)
M1Exo	120.1 ± 0.5	−36.32 ± 1.89	-
M1Exo-GEM-DFX	150.9 ± 0.3	−34.30 ± 3.25	6.5 ± 2.3 (GEM) 5.7 ± 1.4 (DFX)

## Data Availability

All data needed to validate the conclusion in this paper are present in the paper. Additional data related to this paper may be request from the authors.
